# Supplementary material for: The Power and Pitfalls of Big Data Research in Obstetrics and Gynecology: A Consumer's Guide
Source: Obstet Gynecol Surv. 2017 Nov 15;72(11):669–82. doi: 10.1097/OGX.0000000000000504 (PMC5704657; doi:10.1097/OGX.0000000000000504)
Supplement: SUPPLEMENTARY MATERIAL [file ogx-72-669-s001.docx]

**Table 1: Summary of Available Observational Data Resources**

|  | Data Resource | Data Contents | | Data Source and Location | Cost | Software Considerations | Potential Uses/Benefits | Limitations |
| --- | --- | --- | --- | --- | --- | --- | --- | --- |
|  |  |  |  | |  |  |  |  |
| 1 | National Ambulatory Medical Care Survey (NAMCS) ^1,2^ | Administrative survey of ambulatory care services | Centers for Disease Control and Prevention, National Center for Health Statistics (https://www.cdc.gov/nchs/ahcd/index.htm | | 1973-2014, Free | SAS/Stata/SPSS | -Identifies patterns of ambulatory medical care utilization in women  -Pregnancy status is identifiable based on survey question  -Contains prescription, diagnosis, and procedure data | -No patient history  -No follow up care data |
| 2 | National Survey on Drug Use and Health (NSDUH) ^3,4^ | Patient Survey of substance use behaviors | Substance Abuse and Mental Health Services Administration (<https://nsduhweb.rti.org/respweb/homepage.cfm>) | | Free | SAS/Stata for 2010-2015, Online Portal Access (not downloadable) prior to 2010 | -Provides snapshot of substances used by women  -Pregnancy status is identifiable based on survey question  -Information on the use selected substances is generally not available elsewhere | -No outcomes data  -No prescription data  -Self-reported measures with fair to excellent reliability |
| 3 | Medicaid Analytic eXtract Data (MAX) ^5,6,7,8^ | Personal-level data on Medicaid eligibility, service utilization and payments | Medicaid and Children’s Health Insurance Program (CHIP)/ Centers for Medicare and Medicaid Services (CMS) via The Research Data Assistance Center (ResDAC) | | 1999-2013 | SAS/STATA | -Identifiable pregnant woman and resulting neonate  -Prescription/ diagnosis/procedure history  -Large population with nationwide coverage of low-income people, disabled people and ethnic minorities  - Information on many rare maternal and neonatal outcomes  - Feasibility to link Vital statistics, NCHS, NHANES, Medicare Current Beneficiary Survey (MCBS) | -No Inpatient medication exposure and OTC data  -No health behaviors unless diagnosed condition  -No gestational time information for pregnancy research  - Observational time is limited for many pregnant women  - Limited to one specific payer  -Validity of maternal, neonatal, and confounding factors varied |
| 4 | Medicare ^7,9^ | Personal-level data on Medicare eligibility, service utilization and payments | Centers for Medicare and Medicaid Services (CMS) via The Research Data Assistance Center (ResDAC) | | 1999-2015 | SAS/STATAs | -Largest coverage of national elderly population  -Identifiers to track health-care-utilization and outcomes across multiple providers | --No Inpatient medication exposure and OTC data  -Limited to elderly population (>65 years old)  -Limited to one specific payer |
| 5 | Truven Health MarketScan Commercial Claims data ^10^ | Personal-level data on different health care plan eligibility, service utilization and payments | Truven Health MarketScan Commercial Claims data ^11^ | | 2005-2016 | SAS/STATAs | -Identifiable pregnant woman and resulting neonate  -Prescription/ diagnosis/procedure history | --No Inpatient medication exposure and OTC data  -No health behaviors unless diagnosed condition |
| 6 | Prescription Drug Monitoring Program^12^ | State database of patient controlled substance history | Each state designates a state agency to contain data/  (www.nascsa.org/rxMonitoring.htm) | | It varies by state | SAS/SPSS/STATA | -Pharmacy record of controlled substance prescription history  -Cash payments included | -Non-controlled substance prescriptions not available  -Accessibility requires permissions, limited to de-identified data for research  -Limited potential to gain access to more than one state’s data  -Delay in reporting varied by states |
| 7 | The Treatment Episode Data Set (TEDS) ^13^ | Substance abuse treatment admissions | Center for Behavioral Health Statistics and Quality, Substance Abuse and Mental Health Services Administration (SAMHSA)/ https://wwwdasis.samhsa.gov/webt/newmapv1.htm | | 1992-present | SAS/SPSS/STATA | -Identifies substances for which patient is undergoing treatment  -Identifies treatment type | -No follow up care after discharge  -No prescription data  -Only useful for patients with documented substance abuse problems  -Not a population-based database |
| 8 | EHR ^14,15^ | Patient record that is  created in digital format in hospitals and ambulatory  environments. | It varies by different vendors | | It varies by different vendors | SAS/SPSS/STATA/Excel | -Complete clinical outcomes information, organizational outcomes, and societal benefits | -Limited to users of the EHR system (likely missing some high risk populations)  -Requires extensive permissions to use this data for research purposes, procedure will vary by location |
| 9 | NHANES^16,17^ | Patient health behaviors survey | National Center for Health Statistics (NCHS)/  http://www.cdc.gov/nchs/nhanes.htm | | No cost information | SAS/SPSS/STATA/Excel | -Complex information of physical and laboratory examinations  -Large database representing the U.S. population of all ages | -Patient self-reported retrospective information suffering recall bias  -Large portion of missing information  -Lack of follow-up with cross-sectional design  - Minority populations are oversampled |
| 10 | National Vital Statistics^18^ | Official records of live births, deaths, fetal  deaths, marriages, divorces, and annulments. | National Center for Health Statistics (NCHS)/ https://www.cdc.gov/nchs/nvss/index.htm | | No cost information | SAS/SPSS/STATA/Excel | -Ongoing data collection and public availability  -Complete coverage of vital events, including birth and death | -Lack of follow-up with cross-sectional design  -Large portions of inaccurate and incomplete data of maternal and neonatal outcomes and varied by states |
| 11 | Pregnancy Risk Assessment Monitoring System (PRAMS) ^2,83^ | population-based surveillance system designed to identify and monitor selected maternal experiences and behaviors | Centers for Disease Control and Prevention, (<https://www.cdc.gov/prams/>) | | 1988-2014, Free (application required) | Microsoft Excel | -focus on pregnant women with a recent delivery  -collect unique maternal health behavior and attitude related information  -collect Mother's knowledge of pregnancy-related health issues  -linkage to birth certificates are possible based on state approval  -Sampling strategy covers most US states in alternating years | -Not all states included in sampling every year  -Revision occurs periodically leads to loss of continuity on certain measures |
| 12 | Healthcare Cost and Utilization Project (HCUP) ^19^ | Ambulatory, Emergency Department, Inpatient discharge data | Agency for Healthcare and Research and Quality (<https://www.ahrq.gov/research/data/hcup/index.html> | | 1988-2014, Price varies by year and database ($20-$750) | SAS/SPSS/STATA/Excel | -The largest encounter-level data with all-payer information  -Including clinical and non-clinical information  -Feasibility to link other data, such as cancer registry and Medicare | - Limited availability of clinical data  -Possible bias from coding inaccuracies  -Inability to show complete episode of care  -Lack of representation of all hospital types  -Lack of information on revenue or cost  -Varied data elements between different states |
| 13 | National Health Interview Survey (NHIS) ^20^ | Survey of health status and utilization of healthcare services/access | Centers for Disease Control and Prevention (<https://www.cdc.gov/nchs/nhis/>) | | 1997-2015, Free | Data in ASCII format, SAS, Stata, SPSS | -Linkage to death certificate data available  -Includes sampling strategies for nationwide representation of non-institutionalized families  -Contains demographic information, self-report health status and behaviors  -Reports of access to care and use of care  -Useful for tracking conditions, epidemiological, or policy outcomes | -Data is self-report  -Several high risk populations excluded from the survey (the incarcerated, hospitalized, institutionalization for mental illness or disability) |
| 14 | Medical Expenditure Panel Survey (MEPS) ^21^ | Survey of patients (health status/behaviors), their employers (care costs), their providers (utilization and costs) | Agency for Healthcare Research and Quality (<https://meps.ahrq.gov/mepsweb/>) | | 1996-2015, Free | Data in ASCII format (input ready for any stats package) | -Data collected from nationwide sampling strategy  -Linkages between patient-reported health status/behavior, employer insurance costs, and healthcare utilization costs via insurance claims and provider reports  -Detailed billing and cost information for all claims and expenditures (including out of pocket costs, premiums, employer contributions, payment type, insurer type)  -pregnancy status identifiable | -Limited diagnostic/medical detail  -Short follow up period for individuals (maximum respondent participation time is 2 years) |
| 15 | New Mexico Human Papilloma Virus Pap Registry ^22^ (NM HPV PR) | Registry of cervical cancer screenings | Center for HPV Prevention (<http://hpvprevention.unm.edu/NMHPVPR/>) | | 2006-2014, Unknown Cost | Unknown | -Contains linked surveillance data for cervical cancer screenings  -Contains administrative data from MCOs | -Requires approval from registry management, (contact info available at site link) |
| 16 | National ART Surveillance System (NASS) ^23^ | Surveillance data from Assisted Reproductive Technology (ART) clinics | CDC National Center for Chronic Disease Prevention and Promotion, Division of Reproductive Health (<https://www.cdc.gov/art/nass/accessdata.html>) | | 2004-2015, Free | Accessible by CDC terminal location only or through approved data extracts prepared by CDC in user-choice format | -Contains patient characteristics for all undergoing ART cycles in the United States  -Contains procedure information  -Contains patient outcomes after ART | -Requires application and research proposal (instructions at site link)  -Data accessible only through CDC-RDC terminal location after approval or through selective conditions for remote access |
| 17 | States Monitoring Assisted Reproductive Technology (SMART) ^24^ | Collection of state-level surveillance data from ART patients (maternal and infant), linkages with state birth certificates/vital records | CDC National Center for Chronic Disease Prevention and Promotion, Division of Reproductive Health (<https://www.cdc.gov/art/smart/>) | | Florida 1998-2010, Massachusetts 1997-2010, Michigan 1996-2009, Free | None- collaboration with CDC SMART researchers required | -Linked patient (maternal and neonate) data from surveillance to state vital statistics, discharge, and birth certificates for all ART patients in Connecticut, Massachusetts, Florida, and Michigan | -Requires direct approval and collaboration with SMART researchers, data not accessible for private download  -Not all states have linkages or are participating |
| 18 | National Abortion Surveillance^25^ | Aggregated abortion services utilization data from state central health agencies | CDC, Division of Reproductive Health (<https://www.cdc.gov/reproductivehealth/data_stats/abortion.htm>) | | 2009-2013, Free | Microsoft Excel or raw .csv | -Contains trends in utilization for abortion-related procedures  -Some patient demographic characteristics included  -Calculated trend data available as direct “cross tab” downloads from the CDC from 1969 | -State-level participation in reporting varies over years (some states omit reporting fields)  -Medical histories and records for patients not available |
| 19 | Pregnancy-Related Mortality Surveillance System (PMSS) ^26^ | Aggregated state death certificate data- maternal and fetal | CDC, Division of Reproductive Health, Maternal and Infant Health Branch (<https://www.cdc.gov/reproductivehealth/maternalinfanthealth/pmss.html>) | | 1987, 2013, unknown | None- collaboration with Maternal and Infant Health Branch required | -Aggregate reporting of mortality statistics for maternal and fetal pregnancy-related mortality | -Data only available within trend reports unless direct collaboration with CDC |
| 20 | Pelvic Floor Disorders Registry (PFDR) ^27^ | Patient and physician reported outcomes in patients treated for pelvic organ prolapse via surgical or non-surgical procedures | American Urogynecologic Society (https://www.augs.org/clinical-practice/pfd-research-registry/) | | 2013-2016, Free | User choice | -Quality of life metrics, adverse events, and procedure details from patients undergoing pelvic organ prolapse treatment  -Includes follow up treatment data for 36 months after procedures  -Nationwide cohort from multiple treatment sites available | -Research proposal required to access the data  -Unknown participation statistics  -Limited to patient population with one condition |
| 21 | Society of Gynecologic Oncology Clinical Outcomes Registry (SGO COR) ^3^ | Database of diagnoses, treatment procedures, and outcomes for patients with gynecologic cancers | Society of Gynecologic Oncology (<https://www.sgo.org/quality-outcomes-and-research/sgo-clinical-outcomes-registry-2/>) | | 2014-Present, Membership fee varies | SQL extract Limited Data Set files for research | -Patient discharge data, admissions data, birth/death dates, zip codes  -Operative and Post Op 30 day follow up data  -Surveillance and follow up data for chemo/radiation patients  -Institutional data from EHR extracts (member-provided) | -SGO membership required  -Approval required for research, but no approval required for quality improvement metric reporting |
| 22 | American Hospital Association Annual Survey (AHAS) ^28^ | Health system performance data (readmissions, quality metrics, patient satisfaction, mortality) | American Hospital Association (<http://www.ahadata.com/data-collection-methods/>) | | 1980-Present, Price varies based on research needs | Unknown | -Various geographical identifiers linking to other resources  -Ideal for etiological study  -Largest coverage of more than 6,400 hospitals. | -The unit of analysis is hospital and no patient-level of maternal and neonatal information  -Inaccuracies and inconsistencies in reporting  -Low response rates to certain data items-  -Self-reported information from hospital suffering information biases  -A lack of publicly available technical documentation concerning the statistical methodology of the survey |
| 23 | American Medical Association Physician Masterfile^29^ | Database of physician specialties and location | American Medical Association (<https://www.ama-assn.org/life-career/ama-physician-masterfile>) | | 1906-Present, Contact for pricing | Unknown | -Contains all physician credentials and practice locations licensed in the US | -May only be useful for ‘access to care’ studies or analysis of physician credential/specialty trends |
| 24 | Area Health Resource Data^30^ | Health facilities, health professions, measures of resource scarcity, health status, economic activity, health training programs, and socioeconomic and environmental characteristics. | The AHRQ Multiple Chronic Conditions Research Network/  https://www.icpsr.umich.edu/icpsrweb/AHRQMCC/studies/34043 | | It varies by the database | SAS/SPSS/STATA/Excel | -Various geographical identifiers linking to other resources  -Containing more than 50 databases providing contextual geographically-based information  -Ideal for etiological study | -No patient-level of maternal and neonatal information  - Years for which database varied and no longitudinal information |
| 25 | Surveillance, Epidemiology, and End Results Program (SEER) ^7^ | Patient demographics, primary tumor site, tumor morphology and stage at diagnosis, first course of treatment, and follow-up for vital status. | National Cancer Institute/ https://seer.cancer.gov/ | | No cost information |  | -The largest population-based database includes stage of cancer and patient survival information  -Complete and valid data on treatments with surgery and radiation therapy  -Linked to Medicare | -No chemotherapy information  -No information for comorbidities, socioeconomic status, health insurance information, and cause of death |
| 26 | Sentinel Distributed Database^31^**^,^**^32^ | De-identifiable electronic data including patient enrollment demographics, medical utilization, pharmacy prescriptions, diagnoses, laboratory tests and vital signs | The U.S. Food and Drug Administration's (FDA)/ https://www.sentinelinitiative.org/ | | No cost information |  | -It minimizes the need to share identifiable patient information.  -It is an active surveillance system to understand drug and vaccine safety issues  -It accelerates the effectiveness of decision-making progress | -The usability of laboratory data is limited  -It might lose some data integrity under its infrastructure  -The database is under development |

Reference List

1. NAMCS/NHAMCS - Ambulatory Health Care Data Homepage. https://www.cdc.gov/nchs/ahcd/index.htm. Accessed June 16, 2017.

2. Statistics NC for H, others. *Survey Content for the National Ambulatory Medical Care Survey and National Hospital Ambulatory Medical Care Survey*.; 2007.

3. National Survey on Drug Use and Health (NSDUH). https://nsduhweb.rti.org/respweb/homepage.cfm. Accessed June 16, 2017.

4. Chromy JR, Feder M, Gfroerer J, et al. Reliability of key measures in the National Survey on Drug Use and Health. *US Dep Health Hum Serv Subst Abuse Ment Health Serv Adm Off Appl Stud Rockv MD*. 2009.

5. Palmsten K, Huybrechts KF, Mogun H, et al. Harnessing the Medicaid Analytic eXtract (MAX) to Evaluate Medications in Pregnancy: Design Considerations. *PLOS ONE*. 2013;8(6):e67405. doi:10.1371/journal.pone.0067405.

6. Crystal S, Akincigil A, Bilder S, et al. Studying Prescription Drug Use and Outcomes With Medicaid Claims Data Strengths, Limitations, and Strategies. *Med Care*. 2007;45(10 SUPL):S58-S65. doi:10.1097/MLR.0b013e31805371bf.

7. Leonard CE, Brensinger CM, Nam YH, et al. The quality of Medicaid and Medicare data obtained from CMS and its contractors: implications for pharmacoepidemiology. *BMC Health Serv Res*. 2017;17. doi:10.1186/s12913-017-2247-7.

8. Ray WA. Policy and Program Analysis Using Administrative Databases. *Ann Intern Med*. 1997;127(8_Part_2):712. doi:10.7326/0003-4819-127-8_Part_2-199710151-00055.

9. Overview of North American Databases - Mann’s Pharmacovigilance - Strom - Wiley Online Library. http://onlinelibrary.wiley.com/doi/10.1002/9781118820186.ch25/summary. Accessed June 16, 2017.

10. Truven Health Analytics. Marketscan Research DBs | Truven Health Analytics. Truven Health Analytics. https://truvenhealth.com/your-healthcare-focus/analytic-research/marketscan-research-databases. Published 2017. Accessed August 14, 2017.

11. Schneeweiss S, Avorn J. A review of uses of health care utilization databases for epidemiologic research on therapeutics. *J Clin Epidemiol*. 2005;58(4):323-337. doi:10.1016/j.jclinepi.2004.10.012.

12. Griggs CA, Weiner SG, Feldman JA. Prescription Drug Monitoring Programs: Examining Limitations and Future Approaches. *West J Emerg Med*. 2015;16(1):67-70. doi:10.5811/westjem.2014.10.24197.

13. TEDS-Treatment Episode Data Set. https://wwwdasis.samhsa.gov/webt/information.htm. Accessed June 6, 2017.

14. Alpert. The electronic medical record in 2016: Advantages and disadvantages. http://www.digitmedicine.com/article.asp?issn=2226-8561;year=2016;volume=2;issue=2;spage=48;epage=51;aulast=Alpert. Accessed June 7, 2017.

15. Menachemi N, Collum TH. Benefits and drawbacks of electronic health record systems. *Risk Manag Healthc Policy*. 2011;4:47-55. doi:10.2147/RMHP.S12985.

16. Li C, Balluz LS, Ford ES, et al. A comparison of prevalence estimates for selected health indicators and chronic diseases or conditions from the Behavioral Risk Factor Surveillance System, the National Health Interview Survey, and the National Health and Nutrition Examination Survey, 2007-2008. *Prev Med*. 2012;54(6):381-387. doi:10.1016/j.ypmed.2012.04.003.

17. Nelson DE, Powell-Griner E, Town M, et al. A comparison of national estimates from the National Health Interview Survey and the Behavioral Risk Factor Surveillance System. *Am J Public Health*. 2003;93(8):1335-1341.

18. Northam S, Knapp TR. The Reliability and Validity of Birth Certificates. *J Obstet Gynecol Neonatal Nurs*. 2006;35(1):3-12. doi:10.1111/j.1552-6909.2006.00016.x.

19. Abilez C. Evaluation of Health Care Cost and Utilization Project Data (HCUP) in Healthcare Research. *Theses Diss*. May 2013. http://digitalcommons.hsc.unt.edu/theses/132.

20. NHIS - Data, Questionnaires and Related Documentation. https://www.cdc.gov/nchs/nhis/data-questionnaires-documentation.htm. Accessed June 16, 2017.

21. Medical Expenditure Panel Survey Home. https://meps.ahrq.gov/mepsweb/. Accessed June 16, 2017.

22. About the New Mexico HPV Pap Registry :: Center for HPV Prevention | The University of New Mexico. http://hpvprevention.unm.edu/NMHPVPR/. Accessed June 16, 2017.

23. Accessing National ART Surveillance Data | Assisted Reproductive Technology (ART) | Reproductive Health | CDC. https://www.cdc.gov/art/nass/accessdata.html. Accessed June 16, 2017.

24. Mneimneh AS, Boulet SL, Sunderam S, et al. States Monitoring Assisted Reproductive Technology (SMART) Collaborative: Data Collection, Linkage, Dissemination, and Use. *J Womens Health*. 2013;22(7):571-577. doi:10.1089/jwh.2013.4452.

25. Abortion | Data and Statistics | Reproductive Health | CDC. https://www.cdc.gov/reproductivehealth/data_stats/abortion.htm. Accessed June 16, 2017.

26. Pregnancy Mortality Surveillance System | Pregnancy | Reproductive Health | CDC. https://www.cdc.gov/reproductivehealth/maternalinfanthealth/pmss.html. Accessed June 16, 2017.

27. PFD Research Registry - AUGS Registries | AUGS. https://www.augs.org/clinical-practice/pfd-research-registry/. Accessed June 16, 2017.

28. The American Hospital Association’s Annual Survey of Hospitals: a critical appraisal. ResearchGate. https://www.researchgate.net/publication/243464109_The_American_Hospital_Association’s_Annual_Survey_of_Hospitals_a_critical_appraisal. Accessed June 9, 2017.

29. Physician Masterfile | AMA. https://www.ama-assn.org/life-career/ama-physician-masterfile. Accessed June 16, 2017.

30. Asche CV. *Applying Comparative Effectiveness Data to Medical Decision Making: A Practical Guide*. Springer; 2015.

31. Platt R, Carnahan RM, Brown JS, et al. The U.S. Food and Drug Administration’s Mini-Sentinel program: status and direction. *Pharmacoepidemiol Drug Saf*. 2012;21:1-8. doi:10.1002/pds.2343.

32. Sentinel System. https://www.sentinelinitiative.org/. Accessed June 17, 2017.
