# Supplementary material for: The Power and Pitfalls of Big Data Research in Obstetrics and Gynecology: A Consumer's Guide
Source: Obstet Gynecol Surv. 2017 Nov 15;72(11):669–82. doi: 10.1097/OGX.0000000000000504 (PMC5704657; doi:10.1097/OGX.0000000000000504)
Supplement: SUPPLEMENTARY MATERIAL [file ogx-72-669-s002.docx]

**fThe Power and Pitfalls of Big Data Research in Obstetrics & Gynecology:**

**A Consumer’s Guide**

Amie Goodin, PhD, MPP^1^; Chris Delcher, PhD, MS^2^; Chelsea Valenzuela^3^; Xi Wang, MPH, BM^4^; Yanmin Zhu, BA^5^; Dikea Roussos-Ross, MD^6^; Joshua Brown, PharmD, PhD^7^

^1^ Postdoctoral Associate, University of Florida College of Pharmacy, Department of Pharmaceutical Outcomes & Policy.

^2^ Assistant Professor, University of Florida College of Medicine, Department of Health Outcomes & Policy.

^3^ Student, University of Florida, College of Medicine, Department of Health Outcomes & Policy.

^4^ Graduate Student, University of Florida College of Pharmacy, Department of Pharmaceutical Outcomes & Policy.

^5^ Graduate Student, University of Florida College of Pharmacy, Department of Pharmaceutical Outcomes & Policy.

^6^ Assistant Professor, University of Florida College of Medicine, Department of Obstetrics & Gynecology, Department of Psychiatry.

^7^ Assistant Professor, University of Florida College of Pharmacy, Department of Pharmaceutical Outcomes & Policy.

**Corresponding Author:** Amie Goodin, PhD, MPP

1225 Center Drive

University of Florida College of Pharmacy

Gainesville, FL 32610

Email: [amie.goodin@ufl.edu](mailto:amie.goodin@ufl.edu)

Phone: (606) 499-0940

Fax: (352) 273-6270

**Conflict of Interest and Funding Sources:** The authors have no conflicts of interest to declare. No funding was acquired for the completion of this project.

**ABSTRACT**

**Importance:** Research in obstetrics and gynecology (OB/GYN) increasingly relies upon “big data” and observational study designs. There is a gap in practitioner-relevant guides to interpret and critique such research.

**Objective:** This guide is as an introduction to interpreting research using observational data and provides explanations and context for related terminology. Additionally, it serves as a guide for critiquing OB/GYN studies that use observational data by outlining how to assess common pitfalls of experimental and observational study designs. Lastly, the piece provides a compendium of observational data resources commonly used within OB/GYN research.

**Evidence Acquisition:** Review of literature was conducted for the collection of definitions and examples of terminology related to observational data research. Data resources were collected via web-search and researcher recommendations. Next, each data resource was reviewed and analyzed for content and accessibility. Contents of data resources were organized into summary tables and matched to relevant literature examples.

**Results:** We identified twenty-six observational data resources frequently used in secondary analysis for OB/GYN research. Cost, accessibility considerations for software/hardware capabilities, and contents of each data resource varied substantially.

**Conclusions and Relevance:** Observational data sources can provide researchers with a variety of options in tackling their research questions related to OB/GYN practice, patient health outcomes, trends in utilization of medications/procedures, or prevalence estimates of disease states. Insurance claims data resources are useful for population-level prevalence estimates and utilization trends, while EHR-derived data and patient survey data may be more useful for exploring patient behaviors and trends in practice.

**Key Words:** Observational Study, Study Designs, Big Data Research, Obstetrics & Gynecology Research Terminology Guide

**Target Audience**: Obstetricians and gynecologists, family physicians

**Learning Objectives**

**After completing this activity, the learner will be better able to:**

- Identify and define terminology used in observational data research.
- Compare the features, strengths, and limitations of observational study designs and randomized controlled trials.
- Distinguish between types of observational data (e.g., insurance administrative claims, discharges, Electronic Health Record databases, surveys, surveillance data), and weigh the strengths and limitations of research that uses each data type.
- Interpret and critique OB/GYN research that uses observational data and secondary data analysis.
- Gain exposure and familiarity with a selection of observational datasets used to study topics relevant to obstetrical and gynecological practice and/or health outcomes.

**INTRODUCTION**

The provision, administration, and evaluation of contemporary healthcare services generate an enormous quantity of accessible data for research. Access to “big data” by clinicians and researchers increases every year, with tools such as Electronic Health Records (EHRs) and Open Access publication of research data and findings gaining rapid availability. ^1^

Big data in healthcare often refers to EHR databases, patient registries, and administrative claims, among others. These data sources are also employed in a variety of observational research study designs and clinical specialty areas. Research using these data may be undertaken by medical students or residents, junior investigators, or experienced research teams with varying levels of experience and knowledge using these types of data and study design approaches. This “consumer guide” will prepare readers to navigate and interpret research using observational data while providing explanations and context for related terminology. A second objective of this guide is to help the reader, either a junior investigator or an experienced clinician, critique studies in obstetrics and gynecology (OB/GYN) that use observational data, which will be accomplished by outlining how to assess common pitfalls of quasi-experimental study designs that use observational data. The guide then demonstrates the application of this knowledge using a hypothetical case study of an OB/GYN research article using observational data and a quasi-experimental, or non-randomized, design. Finally, a compendium of observational data resources commonly used within OB/GYN research is provided.

**METHODS**

Terminology and definitions relevant to observational data research were identified via literature and keyword searches for terms using PubMed/MEDLINE and Google Scholar. Examples of the relevant identified terminology were based on reviews of the literature in OB/GYN-research focused journals. Categories of observational data were defined and reported in summary tables, with examples of studies using each type of data.

Additional web-searches were conducted to identify data resources and the identified government-sponsored data collection agencies were cross-referenced to find further data resources. Researcher recommendations were also used to gather observational data sources, along with review of current literature for observational studies or secondary data analysis in OB/GYN-focused journals. The contents of each data resource were summarized and the cost and accessibility of each resource was verified with the data provider or government data collection agency. A flow chart was constructed to allow the user to quickly interpret the study design for a quasi-experimental study design using observational data.

**RESULTS**

Review of the literature resulted in the identification of 22 frequently used terms relevant to interpreting observational data research (Table 1). These terms will be used throughout the guide.

*Selecting the Appropriate Study Design*

The reader should use this section to aid in distinguishing quasi-experimental designs using observational data from randomized controlled trials (RCTs) or other experimental designs. Experimental studies, such as RCTs, rely on an investigator-assigned exposure whereas an observational study collects or reviews data from an extant phenomenon or occurrence, that is, the exposure is assigned “naturally” either by clinical decision-making processes, policy changes, etc. (see Figure 1). Figure 2 provides the reader with a “checklist” for quickly interpreting observational data research and will be consulted in later discussion to aid in the critique of an example study abstract.

For OB/GYNs, primary data collection and the use of RCTs is more appropriate for answering research questions such as determining whether a newly developed treatment or medication has the intended effect on patients. This type of research question requires a demonstration of causality, which can more strongly inferred from RCTs due to the use of randomization, and is most often referred as an evaluation of “efficacy,” or answering the question of “Can this work?” Randomization of the “exposure” (for this type of research question the exposure is the treatment or medication) reduces or eliminates the possibility of selection bias when determining what groups in the study population receive the treatment and what groups are used for comparison (e.g., no treatment, placebo, or alternative treatment). This process of randomization allows the investigator to rule out observed effects for reasons other than the treatment. Randomization is thus a crucial component in the investigation of new medications and medical devices, which is why the United States Food and Drug Administration requires the use of RCT study designs during the approval process. RCTs maximize internal validity, i.e. lack selection bias, but have limited external validity due to strict inclusion/exclusion criteria that limit the most sick patients and patients at extremes of age (very young or very old) and operate in a clinical environment that is not comparable to everyday practice. ^2^ As such, many research questions can be left unanswered in underrepresented or disparate patient groups and must be answered using “real-world” data and observational study designs.

OB/GYNs also encounter research questions that may be preferable to investigate using observational study designs or secondary data analysis. Observational data are generated in the real-world either through active data collection for research purposes or passive archiving of data for administrative purposes that can be reused. “Real-world” data are the product of everyday medical practice and not tightly controlled like that of an RCT. Patient medical treatments are part of a complex decision-making process and are non-randomized. Patients will receive treatments based on evaluation of risk factors, comorbid conditions, medical history, behavioral aspects, and other factors. Thus, there are significant threats to validity (i.e., confounding and bias), associated with patient treatment and outcomes that have to be factored into study design and statistical analysis. However, observational data have strong external validity in the population from which they were generated and offer a relatively inexpensive and time-efficient means to conduct clinical research.

For example, it would be impractical, expensive, and potentially impossible to calculate the prevalence of cervical cancer in the general population by performing a cervical cancer screening and medical chart review for every female. We can, however, estimate cervical cancer prevalence using a variety of observational study design methods and data sources, such as; querying patient registries, isolating claims for cervical cancer screenings and treatments in administrative data, surveying patients or clinicians or health systems, or conducting a secondary data analysis of reported surveillance data from government agencies. As another example, an Institutional Review Board would be hesitant to allow an RCT to compare birth defects caused by anti-depressant use in pregnant women due to ethical and liability concerns. However, pregnant women often receive anti-depressants in routine clinical practice and could be evaluated using observational data from a claims database linked to vital statistics. Observational data could then allow for a simultaneous comparison of all anti-depressants and active comparator treatment groups could be factored in to provide stronger evidence. Thus, confounding and bias concerns could be mitigated, for example, by strong study design and/or statistical adjustments using techniques such as regression, propensity score matching, or weighting. Such studies could be conducted in a timely manner using existing resources and without posing direct risk to patients.

*Types of Observational Data*

Seven types of observational data categories were identified from literature searches (Table 2), and each has several strengths and limitations. The summary definitions for these data types (surveys, admissions/discharge data sets, administrative claims data, registries, surveillance data, electronic medical records, and linked data sets) are found in Table 2, with further discussion below. The strengths and limitations with the measurement of exposures, outcomes, and other explanatory factors are discussed below for each of the seven types of observational data categories.

Surveys

“Surveys” constitute an entire family of investigatory tools that sample individual patients, clinicians, or health systems using questionnaires or interviews about a topic defined by the investigator. Sampling strategy, survey instrument design, respondent participation rates, and level of reporting subjectivity are all critical elements of interpreting observational study designs that use surveys. For these reasons surveys tend to be one of the more complicated types of observational data to collect and interpret, and the resulting data are subject to varying levels of both validity and reliability. Secondary data analysis using publicly available survey data sources (e.g., see NAMCS, NSDUH, and NHIS in Supplemental Table 1, Supplemental Digital Content, <http://links.lww.com/OBGYNSURV/A28>) tends to be a better choice for OB/GYN investigators seeking to analyze survey data that was collected in a methodologically rigorous manner. These surveys are often weighted so that survey respondents, once the weights are applied in the statistical analysis, are representative of the general population. Survey data like those collected by various government agencies are collected yearly and also provide a useful source of data to track trends across time in the whole U.S. population. These publicly available sources of survey data, however, come with limitations. None of the examples above were collected explicitly in populations relevant to OB/GYN research, though gender, sex, pregnancy status, and type/frequency of medical visits are available in each of these data resources. These surveys may not be appropriate for rare conditions or very restrictive patient populations as these may not have adequate sample sizes for study even with survey weights applied. Additionally, survey responses may rely on unreliable self-reports and may not include data points relevant to your research question.

Administrative Claims Data

In recent years, large health care administrative claims databases have been widely accepted for research in Obstetrics & Gynecology. Administrative data are derived from automated electronic recording of encounters with the health care system, including physician’s office visits, hospitalizations, filled prescriptions, and diagnostic procedures, which are collected for administrative or billing purposes. ^3,4^ These databases are readily available and relatively inexpensive to purchase/access, and contain clinical information coded using accepted coding systems. They are also representative of routine clinical care and large populations. ^4,5^ Claims data are usually generated from a single insured population (e.g., Medicare, Medicaid, or private insurance), and the generalizability or external validity of research findings outside these discrete populations must be considered. Because administrative claims data are generated primarily for payment and administration of health services, these data are subject to inherent limitations as they are not collected for research purposes. For example, most administrative databases contain limited clinical information. ^5^ Physiological measurements such as blood pressure and glucose levels, tumor stage, etc. are generally not captured. Further, diagnosis and procedure information may not be comprehensive enough, have low sensitivity and specificity for the condition that are meant to capture, and certain chronic conditions could be under-diagnosed. Additionally, the reliability of information in administrative databases largely depends on its impact on payment and can differ between fee-for-service or capitated insurance benefits. ^3^ Treatment and services may not be reflected in the database if they are not covered by health insurance, like elective cosmetic procedures or over-the-counter medications or are simply paid in cash by the patient or administered for free (e.g., vaccines or $4 generic prescriptions). The practice of “upcoding” of patients in order to increase billing has been described and can be a major limitation to research – as well as constituting fraud. ^6^ When evaluating a study using administrative databases, it is imperative to recognize the strengths as well as the limitations of these data.

Despite its limitations, administrative claims databases have a wide spectrum of research applications. Using administrative claims data allows for the study of large patient populations, including pregnant women and infants/children, who tend to be partially or totally excluded from clinical trials. ^4^ Specifically, claims can be used to (1) examine drug utilization patterns (e.g., the prevalence and duration of medication use during pregnancy), (2) study rare events (e.g., breast cancers and congenital malformations), (3) evaluate appropriate prescribing (e.g., antibiotic use and misuse among infants), and (4) link to other resources to expand the use of information in those databases (e.g., linkage to birth certificate data and the National Death Index). Claims data are becoming more widely used to conduct comparative effectiveness and safety research, which generally aims to compare alternative treatments where RCTs may never be performed or may be unfeasible. The U.S. Food and Drug Administration’s Sentinel Initiative is such an effort, developed as an active surveillance network built using a consortium of claims data providers. Claims data can be supplemented by linked data or be used to supplement RCTs or patient registries, which could provide a substantial number data fields for further study or could extend the follow-up period beyond what was originally defined for a clinical study due to budgetary or other constraints.

Admissions/Discharge Data Sets

Health system admission or discharge data sets typically include diagnoses, procedures, and patient demographic characteristics from the hospital or healthcare facility in which the data was collected. These data are similar in format and content to administrative claims data and are thus subject to similar strengths and limitations. Discharge data are typically a one line, cross-sectional record of a hospitalization and are agnostic to when diagnoses and procedures occurred during the hospitalization. One important difference from administrative claims data is that admissions/discharges may include patients that have one or more types of insurance (or that have no insurance), which is typically not captured in administrative claims data that are derived from a single payer source. OB/GYN research that uses discharge data have found that some recorded procedures and diagnoses were highly accurate upon validation study for some conditions, but not as accurate for others. ^7^ The most commonly used discharge database, the National Inpatient Sample, is a random sample of hospitalizations that is weighted to be nationally representative but contains no unique patient identifying identification variable. Thus, such results must be interpreted with caution as the unit of analysis is a “hospitalization” rather than a single patient and some patients may be double counted. Such data are reliable for evaluating trends in utilization or costs and have been used to evaluate variation in care quality. Newer data resources, like the National Readmissions Database, includes patient identifiers and would allow one to follow individuals longitudinally in the data to evaluate 30-day readmissions, mortality, or other outcomes.

Registries

Registries are popular tools in OB/GYN research and are built to track a certain type of condition, use of medication/device, or patient within participating health systems. Registries are often useful for establishing counts of observed side effects, outcomes, and providing preliminary evidence for change over time in prevalence or incidence. One limitation is that registries tend to have voluntary enrollment, which means that participants might be inherently different from non-participants. Another is that registries are typically maintained by institutions with limited geographic penetration (e.g., a single health system or professional organization).

Surveillance Data

Surveillance data is typically collected by a government or nonprofit agency to monitor a condition or attribute that is relevant to public health. Because a particular condition or attribute is the focus of data collection, an OB/GYN may encounter limitations in using this data to answer research questions that require access to detailed clinical data. Additionally, these data sources tend to be de-identified, which makes longitudinal tracking and data linkages difficult or impossible.

Electronic Medical Records

Many OB/GYNs have access to the electronic medical records (also known as EMRs or EHRs) used by their respective healthcare institutions. These data contain detailed clinical information and may also contain extended follow-ups and full medical histories. This depth of information is very valuable to OB/GYN research but the use of this data for research purposes requires substantial review and oversight by your Institutional Review Board, and may require substantial time and staffing resources to filter and condense into usable data for analysis. Additionally, the electronic medical records of a single health system are limited to the geographic area and population serviced by the health system as well as the local policies and practices of the hospital and physicians, which limits the generalizability of study results to the population as a whole. Unless part of a fully integrated health system (e.g., Kaiser Permanente, or the Veterans Affairs system), patient visits to other physician offices or admissions to other hospitals may not be captured in the medical record. Development of a medical record that follows a patient, or is easily accessible from a cloud-based system, is an eagerly anticipated development for researchers looking to use medical records as this will further centralize the data for these purposes.

Linked Data Sets

Data linkages serve an important purpose in OB/GYN research, as this is the primary tool for tracking females and progeny within single data sets and across multiple data sources. For example, observational study designs that require the linkage of state Vital Statistics data (birth certificates and death certificates) are a critical tool for calculating maternal and neonate mortality and morbidity. A use of a common identifier is required to conduct a data linkage, which is why data linkages are difficult to conduct using publicly available data sources (these data are typically de-identified). However, some publicly available observational data resources may conduct a linkage by requiring the investigator to employ the services of an honest-broker third party who conducts the linkage and then removes all identifying data. Such policies will vary significantly based on government or institutional policies.

*Observational Data Resources*

Supplemental Table 1 (Supplemental Digital Content, <http://links.lww.com/OBGYNSURV/A28>) contains a summary of observational data resources that are commonly employed in OB/GYN research. This list is not meant to be exhaustive and in some cases a single prominent example was included for brevity (e.g., commercial insurance claims, where a variety of vendors sell or allow access to different sets of administrative claims from commercial insurers).

**DISCUSSION**

Many researchers are engaged in translating big data resources into actionable benefit for healthcare quality and effectiveness. One study using United Kingdom National Health Service administrative data demonstrated that observational study designs using “big data” could effectively be applied to what the author’s called “small data” (e.g., single institution administrative data). ^8^ In the following sections additional pitfalls and benefits of observational data research are discussed, and then a case study is examined so that the reader may apply the terminology and interpretation of study design to evaluating an OB/GYN study abstract.

*Additional Pitfalls of Observational Data Research*

There are several elements of observational research that we have yet to discuss. While the potential power of big data to disseminate findings or to investigate research questions with larger population sizes is apparent, many urge caution regarding the pitfalls of conducting such research using healthcare data. Namely, large-scale collection and “data-mining” (i.e., a series of statistical techniques for filtering large databases to isolate a characteristic or trend of interest sometimes in the absence of a research hypothesis) practices with healthcare data may pose risks related to causal inference, patient privacy, and/or findings may be susceptible to misinterpretation if inappropriate or unfamiliar study designs or statistical techniques are applied. ^9^ For many, the latter is sometimes referred to as the “black box” and is made easy by modern statistical software. In this section we examine some additional pitfalls of working with observational data; namely, interpreting the relevance of effect size (e.g., clinical versus statistical significance), the technology constraints associated with big data research, and the use of data designed for purposes other than research.

Effect Size: Interpreting Clinical vs. Statistical Significance

It is important for OB/GYN professionals to know how to interpret and implement results from observational data in their clinical decision-making. Observational studies rely on statistical analysis, which simply address the acceptance or rejection of statistical hypothesis. Statistically significant differences are determined by the generally accepted level of probability (the “p-level”), and p-values provide guidance with respect to random error due to sampling rather than a true change of outcome between study groups. Readers should consider several assumptions when interpreting statistical significances, such as sample size, or distribution of the population. The power to detect a given effect size, and observe a statistically significant result, is generally a product of sample size and variation. With larger samples available in big data, the likelihood of statistical significance is high. This means that non-chance differences will be reported as “statistically significant” in big data studies, but those differences may be so small that the effect size, or practical difference, has little value for application to clinical practice. In other words, the observed statistical significance from an observational study alone is insufficient to apply in the clinical practice and may be susceptible to misuse by healthcare providers.

The results of an observational study can be statistically significant, but too minimal to be clinically important. However, there is no standard or defined understanding of clinically relevant changes. “Clinical significance” is generally defined as the smallest meaningful change in an observed effect, but this does not establish a standard effect size to be considered as worthwhile by the practitioner to enact a change in clinical care. ^10,11^ There is a large degree of subjectivity in the judgment of clinical significance that could direct the course of patient care because of disparities in patients’ characteristics, differences across healthcare fields, clinical experience, and goals of clinical care. The aware reader should not focus on measures of significance presented in research articles but should evaluate the effect size, and it’s corresponding confidence intervals, to determine if a given effect size is enough to elicit a change in clinical practice. Further, readers should expect both relative effect sizes and absolute effect sizes from research in order to fully appreciate the magnitude of the effect in question.

Let’s examine a hypothetical example for interpreting effect size and statistical significance: a big data observational study finds a statistically significant effect of an intervention to improve patient preventive care. The intervention in our example is a change in insurance coverage, which was enacted by a large managed care organization to fully cover and encourage annual Pap smear examinations. Administrative claims data was reviewed before and after the change in insurance coverage for all adult female patients in the large managed care organization with samples of several thousand patients receiving examinations in a five-year period prior to the change in coverage and several thousand additional patients receiving examinations in the five-year period following the change in insurance coverage. The study reported two major findings: namely, a p-value that demonstrated a statistically significant difference in the proportion of patients in the managed care organization receiving examinations following the insurance change, and an odds ratio with confidence intervals for the likelihood of patients receiving abnormal pap test results following the coverage change.

These two statistical reports represent very different findings with varying applicability to clinical practice. First, the p-value demonstrating a statistically significant difference in the proportion of patients who received pap tests following the change does indicate that more patients received the tests, but does not indicate *how many more* patients (effect size) received those tests. This p-value has limited clinical applicability because it demonstrates neither the size of the intervention effect nor the desirability of that effect (e.g., More frequent pap testing could potentially result in improved patient outcomes in high risk patients, but what about low risk patients? Is more frequent testing worthwhile for the whole population?). Second, let’s suppose that the odds ratio in our example was reported as: the odds of an abnormal pap test result following the insurance change was 1.03 (95% confidence interval: 1.01-1.05), which means that abnormal test results were found 0.03 times more often following the insurance change. These findings do give the reader information about effect size of the intervention (i.e., the odds of getting abnormal test results went up by 0.03 times, or 3%, following the insurance change); however, this result still lacks clinical context. We know that a greater proportion of patients received annual testing following the insurance coverage change, but we do not know if the additional testing resulted in more false positives (also known as Type I errors) or a true capture of additional patients with abnormal test results. It is also unclear whether a 3% change in effect size is large enough to warrant change in practice- this would remain a clinical decision.

This example brings to light another consideration for working with big data and statistics; that of Type I error, or false positives. There are numerous methods for safeguarding against Type I errors while working with exploratory statistical testing, so best practices would indicate that a statistician or methodologist be consulted to ensure that the selected study design and analysis methods are appropriate for the data available to the researcher.

Technology Constraints and Big Data

*“*Big data” is aptly named, in part, because many sources of observational big data are dramatically larger in sample size than what is typically employed in experimental designs (RCTs). For example, a typical RCT conducted in OB/GYN research may enroll a study population of n=200 female patients, whereas an extract of administrative claims from a large commercial or public insurer data set may contain millions of female patients who fit the study inclusion criteria with tens of millions of individual medical or pharmacy billing records. A data set of this size is not easily stored or analyzed using conventional computer hardware and software. A large data set (or database) may require the use of storage servers, querying software, sophisticated statistical analysis software packages, and the assistance of database administrators and/or statisticians to appropriately manage and analyze data of that magnitude. Access to the infrastructure required to work with big data may be cost or time prohibitive for an OB/GYN practicing outside of a research institution environment with established resources. Other data sources mentioned, like surveys or discharge data, can be managed on local PCs and are amenable to commonly used software packages that are not technically restrictive (e.g., SPSS).

*Additional Benefits of Observational Data Research*

In this section we discuss some additional benefits of working with observational data. First, the methods and procedures typically employed in secondary data analysis tend to be highly reproducible. This is especially true when working with publicly available data and when scholarly publications require thorough reporting of the research protocols employed by observational study designs. As an OB/GYN engaged in research, reproducibility is of tantamount importance when confirming or dismissing emerging evidence. Efforts by several independent groups and scientific societies have sought to standardize reporting of observational studies. Readers should be familiar with the “Strengthening the Reporting of Observational Studies in Epidemiology” (STROBE) guidelines as a checklist for reporting observational study methods. ^12^ Other resources are available for evaluating research using administrative claims data specifically. ^13^

Next, the use of extant data sources is a cost-effective strategy for engaging in research activities. A data source that is both publicly available and kept updated with contemporary entries (e.g., annual surveillance data collected by government agencies) is of high value, but has a low cost of access for the investigator. In some cases the purchase of extant data sources (e.g., commercial insurance administrative claims) may be a more cost-effective means to answering research question than collecting primary data for an RCT, which tend to be expensive even when conducted in small populations. Observational data sometimes can allow for research questions to be evaluated that are deemed unethical or risky for approval of a clinical trial but have occurred in the real-world (e.g., birth defects associated with antipsychotic medications). ^14^

Some observational data resources also allow for the use of large population sizes, which are desirable for the high statistical power that reduces uncertainty and produces more precise estimates of observed phenomenon. A well designed, highly-powered “large N” observational study may produce evidence of greater value than an underpowered “small N” RCT in some cases, particularly among populations where it is difficult to conduct RCTs (e.g., pregnant women). To reiterate, however, readers should remain aware of statistical versus clinical significance in studies with large sample sizes.

*Case Study Application: Evaluating an Abstract that Employs Observational Data*

Now that the reader is familiar with terminology, definitions, and evaluation strategies for interpreting observational data research, we will apply the strategies discussed throughout this guide by critically evaluating the following study abstract relevant to OB/GYNs and other clinicians who treat women and/or neonates. The abstract uses observational data to calculate the Incidence of Neonatal Abstinence Syndrome and quantifies access to care differences for pregnant women seeking opioid abuse treatment:

**“Objective:** Incidence of neonatal abstinence syndrome (NAS) is increasing due to the rise in opioid use. Rural states like Kentucky have been disproportionally impacted by opioid abuse, and this study determines NAS burden nationally and in Kentucky while quantifying differences in access to care between Appalachian and non-Appalachian counties.

**Methods:** NAS rates were calculated using National (2013) and Kentucky (2008-2014) National Inpatient Sample discharge data. Births were identified using International Classification of Diseases v9 code 779.5 and live birth codes V30.x-V38.x. Counties were classified as rural, micropolitan, or metropolitan using census data. Proximity analysis was conducted via mapping from ZIP code centroid to nearest opioid treatment facility. Distance to treatment facilities was calculated and then compared using nonparametric testing for counties by rural and Appalachian status.

**Results:** NAS cases tripled from 2008 to 2014 in Kentucky counties, with a 2013 NAS rate more than double the national NAS rate. Rural and Appalachian counties experienced an NAS increase per 1,000 births that was 2-2.5 times higher than urban/non-Appalachian counties, with a greater number of NAS births overall in Appalachian counties. All opioid treatment facility types were further from rural patients than micropolitan/metropolitan patients (*P* < .001), as well as further for Appalachians versus non-Appalachians (*P* < .001, all facility types).

**Conclusions:** NAS burden disparately affects rural and Appalachian Kentucky counties, while treatment options are disproportionately further away for these residents. Policy efforts to increase NAS prevention and encourage opioid abuse treatment uptake in pregnant women should address rural and Appalachian disparities.”

*Note: This abstract is reprinted with permission from the Journal of Rural Health (Publisher: John Wiley and Sons, Inc.; License #: 4151490014858;* Article citation: Brown JB, et al.; 2017*).* ^15^

Using Figures 1 and 2 as a guide, we have enough information from the study abstract to quickly dissect and interpret the study design and findings. First, we can note that the researchers did not assign an exposure and subjects were not randomized into the study, which means that the study design is non- (or quasi-) experimental. Additionally, the study is retrospective and contains two distinct research objectives: to calculate incidence of neonatal abstinence syndrome in births, and to quantify access to care for opioid abuse among pregnant women in a particular state. The design employed to address the two research objectives would best be described as a descriptive, cross-sectional design, due to the lack of comparison or control groups, lack of longitudinal follow-up data of mothers or neonates, and because the data source lacks individual patient identifiers.

Next, the reader should note that administrative discharge data from the state of Kentucky and the National Inpatient Sample (see Supplemental Table 1, Supplemental Digital Content, <http://links.lww.com/OBGYNSURV/A28>, entry 12 for additional information about this data source from the Healthcare Cost Utilization Project family of data sets) were used in this paper. National data are randomly sampled and would be nationally representative once these weights are applied, which can be used to generate estimates of neonatal abstinence syndrome for the entire country. However, these estimates cannot be calculated by state due to the way data are sampled. Kentucky data are complete records for the state. Though, Kentucky has been hit particularly hard by the opioid abuse epidemic, is rural, and has a distinct Appalachian disparate region, and thus, may not be comparable to other states. Noting the use of discharge data to define the patient population and to isolate diagnoses using billing codes is important, because diagnosis codes may not accurately capture all cases of pregnancy, substance abuse, and neonatal abstinence syndrome in these populations. Neonatal abstinence syndrome could be misdiagnosed, not caused by opioids (as was the focus of that research), and can vary in severity, which is not discernible from a single ICD-9 diagnosis code. The aware reader should evaluate whether the authors’ have supported their choice of coding algorithms used either with prior research or with clear acknowledgement of the potential limitations of the research.

The abstract findings report both incidence rates as well as p-values for differences in distance to opioid abuse care facilities. However, the abstract’s method section provides limited information about the statistical analysis tools used to calculate either of these findings in the study population, which could be cause for concern. Without these details it is difficult to assess if the study is appropriately powered and whether the results are clinically meaningful (e.g., how much higher would neonatal abstinence syndrome incidence in the study location [Kentucky] need to be compared to nationwide incidence to convince me it is a real problem?). Other results in this paper show that women in rural areas would be on average 20 miles further away from pregnancy-specific treatment centers than women in metropolitan areas – a statistically significant finding. While 20 miles may be a burdensome drive, is there evidence that this could affect treatment-seeking behaviors? Is it enough to enact policy changes from state or local government? In such situations it is recommended to delve into the manuscript full-text to find the missing information necessary to interpret the results, including references, which put the effect size in relative terms. If this information is unavailable in the full text, exercise significant caution in your interpretation and application of these study findings.

*Conclusion*

Observational data research and big data can provide clinicians and researchers with a variety of options for conducting and interpreting OB/GYN research, with applications ranging from assessing patient health outcomes, identifying trends in utilization of medications or procedures, or for calculating prevalence estimates of disease states. Different types of observational data have varied strengths and limitations. For example, administrative claims data sources are useful for population-level prevalence estimates and utilization trends, while EHR-derived data and patient survey data may be more useful for exploring patient behaviors and trends in practice. In comparison with RCTs, quasi-experimental designs using observational data may afford the study of larger populations in a more cost-effective manner.

**REFERENCES**

1. Murdoch TB, Detsky AS. The Inevitable Application of Big Data to Health Care. *JAMA*. 2013;309(13):1351. doi:10.1001/jama.2013.393.

2. Rothwell PM. External validity of randomised controlled trials: “To whom do the results of this trial apply?” *The Lancet*. 2005;365(9453):82-93. doi:10.1016/S0140-6736(04)17670-8.

3. Cadarette SM, Wong L. An Introduction to Health Care Administrative Data. *Can J Hosp Pharm*. 2015;68(3). doi:10.4212/cjhp.v68i3.1457.

4. Schneeweiss S, Avorn J. A review of uses of health care utilization databases for epidemiologic research on therapeutics. *J Clin Epidemiol*. 2005;58(4):323-337. doi:10.1016/j.jclinepi.2004.10.012.

5. Romano PS, Hussey P, Ritley D. *Selecting Quality and Resource Use Measures: A Decision Guide for Community Quality Collaboratives*. US Department of Health and Human Services, Agency for Healthcare Research and Quality; 2010.

6. Jesilow P. The Effects of Fraud on the Evaluation of Health Care. *Health Care Anal*. 2005;13(3):239-245. doi:10.1007/s10728-005-6452-x.

7. Yasmeen S, Romano PS, Schembri ME, et al. Accuracy of Obstetric Diagnoses and Procedures in Hospital Discharge Data. *Am J Obstet Gynecol*. 2006;194(4):992-1001. doi:10.1016/j.ajog.2005.08.058.

8. Mann RP, Mushtaq F, White AD, et al. The Problem with Big Data: Operating on Smaller Datasets to Bridge the Implementation Gap. *Front Public Health*. 2016;4. doi:10.3389/fpubh.2016.00248.

9. Gonzales JA, Lietman TM, Acharya NR. The Draw(backs) of Big Data. *JAMA Ophthalmol*. 2017;135(5):422. doi:10.1001/jamaophthalmol.2017.0703.

10. Jaeschke R, Singer J, Guyatt GH. Measurement of health status. Ascertaining the minimal clinically important difference. *Control Clin Trials*. 1989;10(4):407-415.

11. Guyatt GH, Osoba D, Wu AW, et al. Methods to explain the clinical significance of health status measures. *Mayo Clin Proc*. 2002;77(4):371-383. doi:10.1016/S0025-6196(11)61793-X.

12. University of Bern. Institute of Social and Preventive Medicine. Strengthening the Reporting of Observational Studies in Epidemiology (STROBE). STROBE Statement. https://www.strobe-statement.org/index.php?id=strobe-home. Accessed August 8, 2017.

13. Motheral B, Brooks J, Clark MA, et al. A Checklist for Retrospective Database Studies—Report of the ISPOR Task Force on Retrospective Databases. *Value Health*. 2003;6(2):90-97. doi:10.1046/j.1524-4733.2003.00242.x.

14. Rubin DB. The Design Versus the Analysis of Observational Studies for Causal Effects: Parallels with the Design of Randomized Trials. *Stat Med*. 2007;26(1):20-36. doi:10.1002/sim.2739.

15. Brown JD, Goodin AJ, Talbert JC. Rural and Appalachian Disparities in Neonatal Abstinence Syndrome Incidence and Access to Opioid Abuse Treatment: Neonatal Abstinence Syndrome: Rural Disparities. *J Rural Health*. July 2017. doi:10.1111/jrh.12251.

16. Harris AD, McGregor JC, Perencevich EN, et al. The Use and Interpretation of Quasi-Experimental Studies in Medical Informatics. *J Am Med Inform Assoc*. 2006;13(1):16-23. doi:10.1197/jamia.M1749.

17. Rowe H, Sperlich M, Cameron H, et al. A Quasi‐experimental Outcomes Analysis of a Psychoeducation Intervention for Pregnant Women with Abuse‐related Posttraumatic Stress. *J Obstet Gynecol Neonatal Nurs*. 2014;43(3):282-293. doi:10.1111/1552-6909.12312.

18. Payne G, Payne J. Secondary Analysis. In: *Key Concepts in Social Research*. 1 Oliver’s Yard, 55 City Road, London England EC1Y 1SP United Kingdom: SAGE Publications, Ltd; 2004. http://methods.sagepub.com/book/key-concepts-in-social-research. Accessed May 23, 2017.

19. Withagen M, Milani A, de Leeuw J, et al. Development of de novo prolapse in untreated vaginal compartments after prolapse repair with and without mesh: a secondary analysis of a randomised controlled trial: Prolapse repair and effect on untreated vaginal compartments. *BJOG Int J Obstet Gynaecol*. 2012;119(3):354-360. doi:10.1111/j.1471-0528.2011.03231.x.

20. Mann CJ. Observational research methods—Cohort studies, cross sectional studies, and case–control studies. *Afr J Emerg Med*. 2012;2(1):38-46. doi:10.1016/j.afjem.2011.12.004.

21. Nygaard I, Rao S, Dawson J. Anal incontinence after anal sphincter disruption: A 30-year retrospective cohort study. *Obstet Gynecol*. 1997;89(6):896-901. doi:10.1016/S0029-7844(97)00119-1.

22. National Cancer Institute. NCI Disctionary of Cancer Terms. In: *NCI Dictionary of Cancer Terms*. ; 2017. <https://www.cancer.gov/publications/dictionaries/cancer-terms?cdrid=286693>.

23. Macleod M, Strachan B, Bahl R, et al. A prospective cohort study of maternal and neonatal morbidity in relation to use of episiotomy at operative vaginal delivery. *BJOG Int J Obstet Gynaecol*. 2008;115(13):1688-1694. doi:10.1111/j.1471-0528.2008.01961.x.

24. Jacobsson B, Ahlin K, Francis A, et al. Cerebral palsy and restricted growth status at birth: population-based case-control study. *BJOG Int J Obstet Gynaecol*. 2008;115(10):1250-1255. doi:10.1111/j.1471-0528.2008.01827.x.

25. Ernster VL. Nested Case-Control Studies. *Prev Med*. 1994;23(5):587-590. doi:10.1006/pmed.1994.1093.

26. Belghiti J, Kayem G, Dupont C, et al. Oxytocin during labour and risk of severe postpartum haemorrhage: a population-based, cohort-nested case-control study. *BMJ Open*. 2011;1(2):e000514-e000514. doi:10.1136/bmjopen-2011-000514.

27. Sample Size. In: *Encyclopedia of Research Design*. 2455 Teller Road, Thousand Oaks California 91320 United States: SAGE Publications, Inc.; 2010. http://methods.sagepub.com/reference/encyc-of-research-design/n396.xml. Accessed May 25, 2017.

28. Scheller NM, Pasternak B, Mølgaard-Nielsen D, et al. Quadrivalent HPV Vaccination and the Risk of Adverse Pregnancy Outcomes. *N Engl J Med*. 2017;376(13):1223-1233. doi:10.1056/NEJMoa1612296.

29. Cross-Sectional Study. In: *Dictionary of Statistics & Methodology*. 2455 Teller Road, Thousand Oaks California 91320 United States of America: SAGE Publications, Inc.; 2005. http://methods.sagepub.com/reference/dictionary-of-statistics-methodology/n457.xml. Accessed May 25, 2017.

30. Becker JL, Milad MP, Klock SC. Burnout, depression, and career satisfaction: Cross-sectional study of obstetrics and gynecology residents. *Am J Obstet Gynecol*. 2006;195(5):1444-1449. doi:10.1016/j.ajog.2006.06.075.

31. Thiese MS. Observational and interventional study design types; an overview. *Biochem Medica*. 2014;24(2):199-210. doi:10.11613/BM.2014.022.

32. Brotherton JM, Fridman M, May CL, et al. Early effect of the HPV vaccination programme on cervical abnormalities in Victoria, Australia: an ecological study. *The Lancet*. 2011;377(9783):2085-2092. doi:10.1016/S0140-6736(11)60551-5.

33. Statistical Power. In: *Encyclopedia of Survey Research Methods*. 2455 Teller Road,?Thousand Oaks?California?91320?United States of America? Sage Publications, Inc.; 2008. http://methods.sagepub.com/reference/encyclopedia-of-survey-research-methods/n550.xml. Accessed June 20, 2017.

34. Stulberg DB, Dude AM, Dahlquist I, et al. Abortion Provision Among Practicing Obstetrician-Gynecologists: *Obstet Gynecol*. 2011;118(3):609-614. doi:10.1097/AOG.0b013e31822ad973.

35. *Ben Hardy Defines Validation*. London; 2017. <http://methods.sagepub.com/video/ben-hardy-defines-validation>.

36. Bowling CB, Greer WJ, Bryant SA, et al. Testing and Validation of a Low-Cost Cystoscopy Teaching Model: A Randomized Controlled Trial. *Obstet Gynecol*. 2010;116(1):85-91. doi:10.1097/AOG.0b013e3181e45a52.

37. Jüni P, Altman DG, Egger M. Systematic reviews in health care: Assessing the quality of controlled clinical trials. *BMJ*. 2001;323(7303):42-46.

38. Smeenk JMJ. The effect of anxiety and depression on the outcome of in-vitro fertilization. *Hum Reprod*. 2001;16(7):1420-1423. doi:10.1093/humrep/16.7.1420.

39. Elamin S, Langhoff-Roos J, Boedker B, et al. Classification of perinatal death in a developing country. *Int J Gynecol Obstet*. 2003;80(3):327-333. doi:10.1016/S0020-7292(02)00380-6.

40. *Time Series Design*. 1 Oliver’s Yard, 55 City Road, London England EC1Y 1SP United Kingdom: SAGE Publications, Ltd; 2006. http://methods.sagepub.com/reference/the-sage-dictionary-of-social-research-methods/n208.xml. Accessed May 25, 2017.

41. Crofts J, Lenguerrand E, Bentham G, et al. Prevention of brachial plexus injury-12 years of shoulder dystocia training: an interrupted time-series study. *BJOG Int J Obstet Gynaecol*. 2016;123(1):111-118. doi:10.1111/1471-0528.13302.

42. van den Hout A, Tom B. Survival Analysis and the Frailty Model. In: *The SAGE Handbook of Multilevel Modeling*. 1 Oliver’s Yard, 55 City Road, London EC1Y 1SP United Kingdom: SAGE Publications Ltd; 2013:541-558. http://methods.sagepub.com/book/the-sage-handbook-of-multilevel-modeling/n30.xml. Accessed May 25, 2017.

43. Hart R, Molnar BG, Magos A. Long term follow up of hysteroscopic myomectomy assessed by survival analysis. *BJOG Int J Obstet Gynaecol*. 1999;106(7):700-705. doi:10.1111/j.1471-0528.1999.tb08370.x.

44. Tooth L. Quality of Reporting of Observational Longitudinal Research. *Am J Epidemiol*. 2005;161(3):280-288. doi:10.1093/aje/kwi042.

45. Hecher K, Bilardo CM, Stigter RH, et al. Monitoring of fetuses with intrauterine growth restriction: a longitudinal study. *Ultrasound Obstet Gynecol*. 2001;18(6):564-570. doi:10.1046/j.0960-7692.2001.00590.x.

46. Blessed WB, Lacoste H, Welch RA. Obstetrician-gynecologists performing genetic amniocentesis may be misleading themselves and their patients. *Am J Obstet Gynecol*. 2001;184(7):1340-1344. doi:10.1067/mob.2001.115049.

47. Nonresponse Bias. In: *Encyclopedia of Survey Research Methods*. 2455 Teller Road, Thousand Oaks California 91320 United States of America: Sage Publications, Inc.; 2008. http://methods.sagepub.com/reference/encyclopedia-of-survey-research-methods/n340.xml. Accessed May 25, 2017.

48. Morgan MA, Hawks D, Zinberg S, et al. What Obstetrician-Gynecologists Think of Preconception Care. *Matern Child Health J*. 2006;10(S1):59-65. doi:10.1007/s10995-006-0086-y.

49. Imputation. In: *Encyclopedia of Survey Research Methods*. 2455 Teller Road, Thousand Oaks California 91320 United States of America: Sage Publications, Inc.; 2008. http://methods.sagepub.com/reference/encyclopedia-of-survey-research-methods/n216.xml. Accessed May 25, 2017.

50. Steinauer J, Landy U, Filippone H, et al. Predictors of abortion provision among practicing obstetrician-gynecologists: A national survey. *Am J Obstet Gynecol*. 2008;198(1):39.e1-e39.e6. doi:10.1016/j.ajog.2007.06.002.

51. Weighted Sample. In: *Dictionary of Statistics & Methodology*. 2455 Teller Road,?Thousand Oaks?California?91320?United States of America? SAGE Publications, Inc.; 2005. http://methods.sagepub.com/reference/dictionary-of-statistics-methodology/n2084.xml. Accessed June 20, 2017.

52. Hladky KJ, Allsworth JE, Madden T, et al. Womenʼs Knowledge About Intrauterine Contraception: *Obstet Gynecol*. 2011;117(1):48-54. doi:10.1097/AOG.0b013e318202b4c9.

53. Post-Stratification. In: *Encyclopedia of Survey Research Methods*. 2455 Teller Road,?Thousand Oaks?California?91320?United States of America? Sage Publications, Inc.; 2008. http://methods.sagepub.com/reference/encyclopedia-of-survey-research-methods/n388.xml. Accessed June 20, 2017.

54. Chung GS, Lawrence RE, Rasinski KA, et al. Obstetrician-gynecologists’ beliefs about when pregnancy begins. *Am J Obstet Gynecol*. 2012;206(2):132.e1-e132.e7. doi:10.1016/j.ajog.2011.10.877.

55. Nielsen PE, Goldman MB, Mann S, et al. Effects of Teamwork Training on Adverse Outcomes and Process of Care in Labor and Delivery: A Randomized Controlled Trial. *Obstet Gynecol*. 2007;109(1):48-55. doi:10.1097/01.AOG.0000250900.53126.c2.

56. Laz TH, Rahman M, Berenson AB. An update on human papillomavirus vaccine uptake among 11–17 year old girls in the United States: National Health Interview Survey, 2010. *Vaccine*. 2012;30(24):3534-3540. doi:10.1016/j.vaccine.2012.03.067.

57. Agency for Healthcare Quality and Research. *The Value of Hospital Discharge Databases*. Bethesda, MD; 2005:102. https://www.hcup-us.ahrq.gov/reports/final_report.pdf. Accessed August 8, 2017.

58. Biggs MA, Harper CC, Malvin J, et al. Factors Influencing the Provision of Long-Acting Reversible Contraception in California: *Obstet Gynecol*. 2014;123(3):593-602. doi:10.1097/AOG.0000000000000137.

59. Muller WJ, Miller ES. Preliminary Results From the US Zika Pregnancy Registry: Untangling Risks for Congenital Anomalies. *JAMA*. 2017;317(1):35. doi:10.1001/jama.2016.18632.

60. Buehler JW, Hopkins RS, Overhage JM, et al. *Framework for Evaluating Public Health Surveillance Systems for Early Detection of Outbreaks: Recommendations from the CDC Working Group*.; 2004. https://www.cdc.gov/mmwr/pdf/rr/rr5305.pdf. Accessed August 8, 2017.

61. Roberson EK, Hurwitz EL. Prescription Drug Use During and Immediately Before Pregnancy in Hawai‘i — Findings from the Hawai‘i Pregnancy Risk Assessment Monitoring System, 2009–2011. *Hawaii J Med Public Health*. 2014;73(12):382-386.

62. Agency for Healthcare Research and Quality. Health Information Technology Archive: Electronic Medical Record Systems. https://healthit.ahrq.gov/key-topics/electronic-medical-record-systems. Accessed August 8, 2017.

63. Loudon H, Nentin F, Silverman ME. Using clinical decision support as a means of implementing a universal postpartum depression screening program. *Arch Womens Ment Health*. 2016;19(3):501-505. doi:10.1007/s00737-015-0596-y.

64. Agency for Healthcare Research and Quality. *Linking Data for Health Services Research: A Framework and Guidance for Researchers*.; 2013. https://www.effectivehealthcare.ahrq.gov/ehc/products/494/1746/registries-linking-records-methods-draft-131107.pdf. Accessed August 8, 2017.

65. Salemi JL, Tanner JP, Bailey M, et al. Creation and evaluation of a multi-layered maternal and child health database for comparative effectiveness research. *J Regist Manag*. 2013;40(1):14-28.

**List of Tables**

Table 1: Useful Definitions in Observational Research

Table 2: Types of Observational Data

Supplementary Table 1: Summary of Available Observational Data Resources (Online version with bibliography and links to data resources)

**List of Figures**

Figure 1: Classifying Study Design

Figure 2: A 4-Step Approach for Identifying and Interpreting Observational Studies
